# A novel Tick Carousel Assay for testing efficacy of repellents on * Amblyomma americanum L.*

**DOI:** 10.7717/peerj.11138

**Published:** 2021-04-21

**Authors:** Hailey A. Luker, Stacy Rodriguez, Yashoda Kandel, Julia Vulcan, Immo A. Hansen

**Affiliations:** Department of Biology, New Mexico State University, Las Cruces, NM, United States of America

**Keywords:** *Amblyomma americanum*, Tick Carousel Assay, DEET, IR3535, Picaridin, Oil of Lemon Eucalyptus, Repellent testing, Novel tick assay, Fabric engagement, Hard ticks

## Abstract

Ticks are important vectors of human and veterinary diseases. A primary way ticks gain access to human hosts is by engaging to clothing. Repellents or acaricides sprayed onto fabric are used to deter ticks’ access to human hosts. However, there are a limited amount of standardized laboratory assays that can determine the potency and efficacy of repellents. We present a novel fabric-engagement assay referred to as the ‘Tick Carousel Assay’. This assay utilizes fabric brushing past ticks located on an artificial grass patch and measures tick engagements to fabric over time. After screening a variety of tick species, we used the lone star tick *(Amblyomma americanum*) to test the efficacy of four commonly used active ingredients in repellents: DEET, Picaridin, IR3535, and Oil of Lemon Eucalyptus. Repellency was tested immediately, after three hours, and six hours post application to fabric. Our data show that each repellent we tested significantly reduced the number of tick engagements to fabric for at least 6 hours. We did not find significant differences in repellent efficacy between the four active ingredients tested directly and three hours after application. After six hours, Oil of Lemon Eucalyptus repelled ticks more than the other active ingredients. We show that our Tick Carousel Assay provides an affordable, repeatable, and standardized way to compare and test repellent efficacy on treated fabrics. Our results confirm that commonly used repellents applied to fabric are an effective way to reduce tick engagement.

## Introduction

Ticks are vectors of pathogenic microorganisms such as bacteria, protozoans, and viruses that cause human and veterinary diseases (Fivaz, Petney & Horak, 2012; Ghosh, Azhahianambi & Yadav, 2007). These vectors pose a threat to the health of outdoor recreationists and people in regions with high tick activity (Salkeld et al., 2019). Transmission of pathogens from these vectors occurs when a tick injects saliva into its host while taking a blood meal (Mccoy, Léger & Dietrich, 2013).

To locate and identify a host, ticks use olfactory cues like carbon dioxide emissions, ammonia, and short organic odorants, as well as visual cues, vibrations, and heat (Osterkamp et al., 1999). Their first pair of legs contain a sensory structure called the Haller’s Organ that detects olfactory cues and heat (Carr et al., 2017; Carr & Salgado, 2019). Host-seeking ticks locate to areas that will increase their chance of contacting a host, such as the end of a grass blade, where some ticks show a behavior known as ‘questing’. Questing is when a tick stretches out its forelegs in an attempt to engage a passing host (Nicholson et al., 2019). Upon engagement, ticks climb on the host and begin to search for a suitable feeding site where they will attach (Anderson, 2002). A tick attaches to its host by cementing itself into the host’s skin. Once attached, the tick will begin to ingest blood and tissue fluids (Kemp, Stone & Binnington, 1982) until engorgement. When a tick is fully engorged it will fall from its host and either molt or lay egg depending on its stage in its life cycle. Each species of tick differs in the host they seek depending on their life stage. Species like *Amblyomma americanum* and *Rhipicephalus sanguineus* feed on a wide range of hosts at all life stages regardless of host size and are observed to seek human hosts at all life stages (Mangan, Foré & Kim, 2018). Other species like *Dermacentor variabilis* seek smaller hosts as nymphs and gradually hunt larger hosts, such as humans, as they molt into adults (Nicholson et al., 2019).

Tick repellents are chemicals that deter the engagement of ticks to their hosts, therefore preventing the transmission of pathogens. Four active ingredients found in commonly used commercial insect repellents have been observed to have tick repellent properties. These active ingredients are DEET, Picaridin, IR3535, and Oil of Lemon Eucalyptus (Bissinger & Roe, 2010; Carroll et al., 2010; Granett & French, 1950; Jaenson, Garboui & Pålsson, 2006; Rodriguez et al., 2017; Staub et al., 2002).

A variety of assays have been developed to test the efficacy of repellents on ticks in standardized experiments (Dautel, 2004). These assays can be grouped into three categories: setups with no attractant stimuli, with attractant stimuli, and with live host stimuli. Assays with no attractant stimuli test repellents by comparing the behavior of a tick in the presence of a repellent to its behavior in the absence of one. A study conducted by Carroll and her colleagues tested repellency of DEET and AI3-37220 on *Ixodes scapularis* and *Amblyomma americanum* through a petri dish assay. In their assay, ticks were placed on an untreated filter paper located in a petri dish treated with repellent along the perimeter of the dish (Carroll et al., 2004). The location of the ticks was recorded. If the ticks remained off the treated locations they were considered to be repelled. The absence of tick host-seeking behavior in these assays limit the ability to test the degree of repellency. Testing the degree of repellency is helpful when comparing repellents.

Attractant stimuli assays, like moving-object, tick feeding, and olfactometer assays, are assays that include an attractant that induces host-seeking behavior in ticks and can be used to test and compare repellents. When using an attractant, the potency of a repellency can be quantified. Dautel and coworkers conducted a repellency study using a moving-object bioassay with field-collected *Ixodes ricinus* nymphs (Dautel et al., 1999). Their bioassay utilized heat and movement to stimulate tick host-seeking behavior. The attraction of ticks to the control and experimental treatments were measured to determine repellency.

Assays that utilize live hosts are another method to test repellency. Laboratory animal host assays, human volunteer assays, and field-testing assays are examples of this category. However, the use of live hosts and human volunteers is highly regulated and can be expensive. Field tests with human volunteers have been used to study the effect of repellent-treated pants on the engagement of ticks (Granett & French, 1950). A disadvantage of field-testing is the many environmental variables that cannot be controlled. These variables, like temperature, humidity, and tick abundance, provide obstacles to reproducing results.

In the current study, a novel moving-object bioassay was developed to demonstrate the efficacy of repellents sprayed on fabric. This bioassay, referred to as the ‘Tick Carousel Assay’, utilizes carbon dioxide emissions, body heat, and moving fabric as attractant stimuli for *Amblyomma americanum*. The Tick Carousel Assay measures the number of tick engagements to pieces of moving fabric in the presence or absence of repellent treatments. This assay can be used to test the efficacy of repellents.

## Materials and Methods

### Ticks

Adult females of three tick species, *Amblyomma americanum*, *Dermacentor variabilis*, and *Rhipicephalus sanguineus*, were obtained from the Oklahoma State University (OSU) Tick Rearing Facility.

### Construction of the carousel

**Figure 1:**
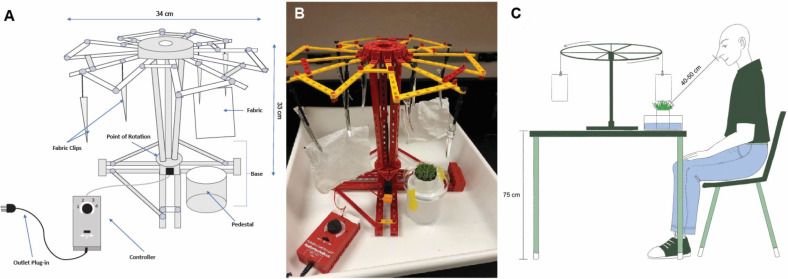
Setup of the Tick Carousel Assay including dimensions and labelled parts. (A) Schematic of the setup with dimensions. (B) Photographic image of the setup. Note the white fabric pieces attached to the clips. (C) Overview of setup including experimenter’s position.

We used a construction kit (fischertechnik: Super Fun Park, Art.-No. 508775; Fischer group, Waldachtal, Germany) to make an electrical-powered merry-go-round model with a diameter of 34 cm and a height of 33 cm (Fig. 1). Two pairs of metal alligator fabric clips were attached to the arms of the carousel, as fabric holders. Using a powered controller (fischertechnik Power Set, Part# 91087) the rotation speed of the carousel can be adjusted.

### Construction of the tick island

**Figure 2:**
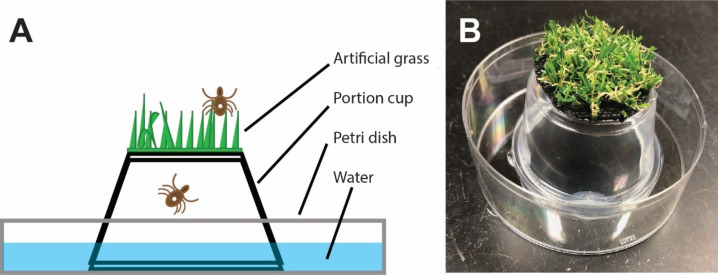
Diagram of tick island used in the Tick Carousel Assay. (A) Schematic of the tick island. (B) Photograph of a tick island without water filling the petri dish.

The tick island was constructed by using a hot melt adhesive and a hot glue gun (Gorilla Glue Co., Cincinnati, OH, USA) to glue an inverted 2oz portion cup with a base diameter of four cm and a top diameter of 6.3 cm (DuraHome, Portion Cup 2oz, item number: 01350, Scripps Co., Cincinnati, OH, USA) into a petri dish with a diameter of 10 cm (Sigma Aldrich, St. Louis, MO, USA, product number: P5731). A circular patch of artificial grass (Turf Factory Direct, Resaca, GA, USA) was cut four cm in diameter and trimmed to have approximately one cm long blades of grass. The patch of artificial grass was glued to the four cm base of the inverted portion cup (Fig. 2). Freshly made islands were stored at room temperature for at least 12 hours before use to allow any fumes produced from the hot melt adhesive or plastic softeners to evaporate.

### Repellent products

Four commercial repellent products were chosen based on their active ingredients (Moore et al., 2018). Table 1 shows the four products tested in this study.

**Table 1:** Repellent products used in this study. Four commercial products with four different active ingredients were chosen. The protectiontimes were taken from the label and refer to skin applications. Insect repellency of thesespecific products has been proven in several studies.

**Commercial product**	**Active ingredient**	**% Active ingredient**	**Protection time (hrs)**	**Citations**
Ben’s^®^ Tick and Insect Repellent	DEET	98.11	10	Brown & Hebert (1997)
REPEL^®^ Plant-Based Lemon Eucalyptus Insect Repellent	PMD	30.00	6	Rodriguez et al. (2015)
SAWER^®^ Premium Insect Repellent 20% Picaridin	Picaridin	20.00	14	Rodriguez et al. (2017)
AVON Skin-So-Soft Bug Guard plus IR3535 EXPEDITION^TM^	IR3535	19.60	8	Rodriguez et al. (2017)

### Tick engagement assay

Experiments were conducted late spring through fall in a well-lit, temperature- and humidity-controlled room. The room was at 27 °C with humidity at 55%. 11.5 cm × 11.5 cm fabric pieces were cut from 33 cm × 33 cm fabric squares (Cotton Pocket Rags, HDX, Home Depot, Atlanta, GA, USA). Treated fabric pieces were sprayed with 0.5 ml of repellent, using a standard spray bottle (85 ul/spray) that was held approximately five cm away from fabric. Three sprays were focused along the bottom of the fabric and three sprays were focused along the center. The treated fabrics were either used immediately or stored for a defined time (3 or 6 hours). Control fabric pieces received no treatment. Two fabric pieces were attached to the carousel using alligator clips. For each run, two pieces with either no treatment (control) or the same experimental treatment were used. The fabric pieces were adjusted to assure fabric brushed over the grass blades of the island. The tick island’s moat was filled with water to prevent ticks from leaving the island (see Supplemental Video). 15 female lone star ticks were transferred to the island using soft #1 featherweight forceps (BioQuip, Rancho Dominguez, CA, USA). The ticks’ questing behavior was induced by the experimenter blowing on the tick island at the beginning of the run and again after 2.5 minutes. When the experimenter was not blowing on the island, their uncovered face remained between 40-50 cm from the tick island. This distance was measured from the experimenter’s nose to the top of the tick island. The experimenter sat in a chair beside the assay during each run (Fig. 1C). The attractant stimuli utilized in the Tick Carousel Assay are carbon dioxide emissions, body heat, and fabric movement. These stimuli were present for the control and treatment runs. The carousel rotated at a speed of 10 rpm for 5 minutes to allow both fabrics a combined 100 passes over the island. Each control run was required to receive at least five engagements before repellent testing could be conducted. A tally counter was used to record tick engagements. If an engaged tick released from the fabric it was returned onto the island. Only engagements were recorded when a tick adhered to the fabric with its tarsi (forelegs). If the tick was brushed off the island by the fabric without engagement, it was not recorded and the tick was returned to the island. We used individual islands for each control and experimental replicate. Islands used for repellent tests were discarded after one use.

### Statistical analysis

All statistical tests were performed using Prism8 (Graph Pad software, San Diego, CA, USA). *P*-value less than 0.05 were considered significantly different. The distribution of the data was graphically inspected using quantile-quantile (Q-Q) plots. Two-way ANOVA was done to analyze the effect of two grouping variables, time and the type of the repellant, on the response variable that is average engagement. Tukey’s post hoc test was done for multiple comparisons. View raw data and statistical data in Supplemental File.

## Results

### Identification of a suitable tick species for the carousel assay

We tested the basic performance of our assay under control conditions on three different tick species: *Amblyomma americanum* (lone star tick)*, Dermacentor variabilis* (american dog tick)*, and Rhipicephalus sanguineus* (brown dog tick). Adult females of each species were tested in the summer. We found that each species varied in behavior and activity when applied to the Tick Carousel Assay. *Amblyomma americanum* proved to be the most aggressive and responsive to our assay, on average seven of 14 ticks would engage the fabric within five minutes. *Dermacentor variabilis* were active and showed questing behavior, however, they negativley responded to the passing fabric by burrowing into the artificial grass, resulting in an average of fewer than two engagements within the five-minute interval. *Rhipicephalus sanguineus* were relatively unresponsive to the attractant stimuli in our assay and showed low activity, compared to the other two species of tick, resulting in an average of zero engagements to fabric within five minutes. We concluded that *Amblyomma americanum* was the best-suited species for the Tick Carousel Assay.

### Efficacy of repellents to deter tick engagement to fabric

**Figure 3:**
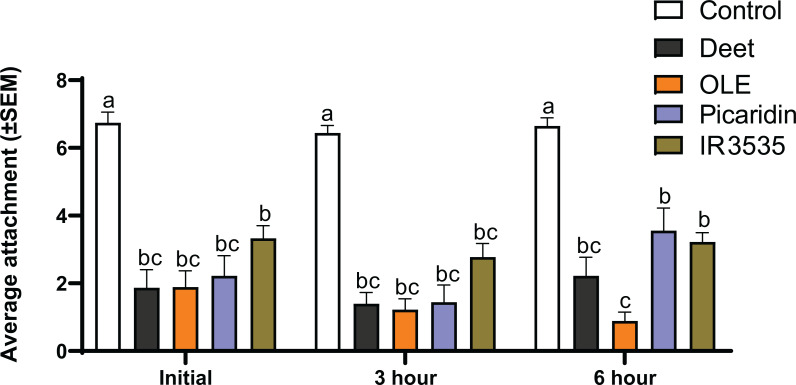
Results from Tick Carousel Assay testing. Shown above is the average number of engagements over a five-minute interval for each trial at three different time points. The bars on the columns represent standard errors. The letters above the columns indicate the results of our statistical analysis. Columns that share the same letter are not significantly different from each other, while columns that do not share a letter are significantly different.

Figure 3 shows the average number of tick engagements at three different time-intervals for control and treated fabrics. Our data shows that the active ingredients DEET, Oil of Lemon Eucalyptus, Picaridin, and IR3535 had a deterring effect on *A. americanum*’s engagement to passing fabric. We found statistically significant differences in engagement rates between the control fabrics and fabrics treated with any of these four active ingredients. All four active ingredients performed equally as tick repellents at the initial and the three hour time point. However, at six hours after application, OLE-treated fabrics had significantly fewer engagements compared with fabric treated with Picaridin or IR3535, but not DEET.

## Discussion

Tick-borne diseases are on the rise in the U.S., putting a large number of people living in tick-endemic areas at risk (Eisen, 2020; Eisen et al., 2017; Paules et al., 2018). Tick repellents are a strategy widely used for individual protection from tick bites (Pages et al., 2014). Standardized laboratory-based methods to test the efficacy of old and new active ingredients and commercial products have the potential to replace field testing, which is subject to many variables that are difficult to control (Dethier, 1956). The Tick Carousel Assay we present here is a novel approach developed to test the efficacy and protection times of tick repellents in an affordable and standardized way. The principle of this assay is ticks are placed on a patch of artificial grass where their host-seeking behavior is triggered by carbon dioxide emissions of an experimenter’s breath. Following the activation of host-seeking, fabric is drawn over the artificial grass and tick engagements to the fabric are recorded. Any efficient repellent applied to the fabric will reduce the number of tick engagements significantly compared to untreated control fabric.

A critical goal at the start of our project was to identify a tick species that was suitable for the carousel assay. We tested three species that showed significant behavioral differences under test conditions. *Amblyomma americanum* showed to be suitable for our assay as they readily engaged the fabric under control experimental conditions. The other two species, *Dermacentor variabilis*, and *Rhipicephalus sanguineus,* did not show this behavior. The pronounced differences in behavioral responses we observed in these tick species are of interest, considering both, *Amblyomma americanum* and the *Dermacentor variabilis*, are commonly reported human-biting ticks (Kessler et al., 2018; St John et al., 2016) while *Rhipicephalus sanguineus* is specialized on dogs and only occasionally bites humans. *Rhipicephalus sanguineus* shows an unusual life cycle, as it spends its whole life on only one host (Dantas-Torres, 2010).

*Amblyomma americanum* and *Dermacentor variabilis* differ in host preference, seasonality, and habitat. *Amblyomma americanum* larvae begin activity in summer, while nymphs and adults start host-seeking behavior during spring (Nicholson et al., 2019). *Amblyomma americanum* adults use a combination of hunting and ambushing to access their host. This species prey on a large variety of terrestrial vertebrates throughout all life stages (Bissinger & Roe, 2010; Kessler et al., 2018; Mangan, Foré & Kim, 2018; Tirloni et al., 2017). In contrast, *Dermacentor variabilis* larval and nymph stages are most active at the end of winter and beginning of spring. At these stages, *Dermacentor variabilis* hunt small mammals and birds. As adults, they experience highest activity during the spring and summer, when they hunt medium to large mammals including dogs and humans (Burgdorfer, 1969; Nicholson et al., 2019). Both species have some overlapping habitats but they are commonly found in different regions (Burgdorfer, 1969). *Amblyomma americanum* inhabit woodlands (Kessler et al., 2018), and *Dermacentor variabilis* inhabit brushy plains (Minigan et al., 2018).

We suggest that the pronounced differences in behavior between the three species we studied might be due to species-specific hunting behaviors and seasonal differences in general activity. It is paramount for the successful establishment of the carousel assay to identify a tick species that ‘cooperates’ with the assay and engages readily to the fabric under control conditions. We observed seasonal differences in the activity of *Amblyomma americanum* during the winter season, however, these anecdotal observations require further research to determine if there are seasonal activity patterns that could interfere with the assay.

We chose four commercially available insect/tick repellent products, that had been evaluated in scientific studies before (see Table 1), for the validation of the Tick Carousel Assay. These four products contained four different active ingredients- DEET, Picaridin, IR3535, and Oil of Lemon Eucalyptus. The first three are effective repellents against *A. americanum* in a human subject experiment where nymphs were challenged to cross a band of repellent-treated skin on a volunteer (Carroll et al., 2010). Oil of lemon eucalyptus showed repellency against *A. americanum* in a two-choice bioassay where the ticks could choose between a treated and an untreated cotton cheesecloth (Bissinger et al., 2009). All four active ingredients have been shown to repel ticks in field studies, some of them using repellent-treated fabrics. In a study conducted by Solburg and collaborators human volunteers who walked through regions of known *Amblyomma americanum* activity and observed numbers of ticks that were contracted on a treated and an untreated control leg (Solberg et al., 1995). They confirmed that DEET and piperidine are effective repellents against *Amblyomma americanum*. They also note seasonal differences in tick activity that made the study more challenging. An example of a field study in which repellent-treated fabric was tested was performed by Schreck, Snoody, and Mount (Schreck, Snoddy & Mount, 1980). They tested permethrin, DEET, and mixture M-1960 - treated military fatigue uniforms worn by subjects. The subjects wore the treated military fatigue uniforms and walked, sat, lay, and stood in highly infested habitats. Infested habitats were determined by control clothing worn in the area contracting 25 or more ticks. Their results concluded that DEET, M-1960 and permethrin each had some level of repellency on *Amblyomma americanum* at all life stages. Our results confirm the results of the aforementioned studies regarding the active ingredients we tested.

In summary, we suggest that the Tick Carousel Assay is a powerful approach to test tick repellents in a controlled laboratory environment. It is a safe, scalable, and effective method to determine the potency and efficacy of new, old, and commercial repellents.

##  Supplemental Information

10.7717/peerj.11138/supp-1 Supplemental Information 1 Raw data from experiments and statistical analysis Table S1 contains the raw data collected for the repellency testing.
Table S2 provides the average tick engagements and standard errors.
Fig. S1 shows a QQ plot of our data normality analysis.

10.7717/peerj.11138/supp-2 Supplemental Information 2 Video of setup and experimental design Beginning of the video: Full view of the Tick Carousel Assay with ticks located on the tick island.
Next: Demonstration of placing ticks on the island before run.
Last: Close up of tick island and fabric passing to observe the engagement of ticks.

## Additional Information and Declarations

### Data Availability

The following information was supplied regarding data availability: Raw data and a video of the assay are available in the Supplemental Files.
