# Supplementary material for: A novel Tick Carousel Assay for testing efficacy of repellents on Amblyomma americanum L
Source: PeerJ. 2021 Apr 21;9:e11138. doi: 10.7717/peerj.11138 (PMC8067905; doi:10.7717/peerj.11138)
Supplement: Supplemental Information 1 — Table S1 contains the raw data collected for the repellency testing. Table S2 provides the average tick engagements and standard errors. Fig. S1 shows a QQ plot of our data normality analysis. [file peerj-09-11138-s001.docx]

|  | **Adult Female Amblyomma Americanum Repellency Tests** | | | | | |
| --- | --- | --- | --- | --- | --- | --- |
| **Time Point** | **0 Hour** | | **3 Hour** | | **6 Hour** | |
|  | **Control** | **Deet** | **Control** | **Deet** | **Control** | **Deet** |
|  | 6 | 2 | 6 | 2 | 7 | 2 |
|  | 9 | 4 | 6 | 0 | 7 | 5 |
|  | 7 | 0 | 7 | 1 | 8 | 1 |
|  | 5 | 0 | 9 | 3 | 8 | 1 |
|  | 6 | 2 | 5 | 3 | 9 | 3 |
|  | 8 | 3 | 7 | 2 | 8 | 1 |
| **Average** | 6.833333333 | 1.833333333 | 6.666666667 | 1.833333333 | 7.833333333 | 2.166666667 |
| **Standard Deviation** | 1.471960144 | 1.602081979 | 1.366260102 | 1.169045194 | 0.752772653 | 1.602081979 |
| **Standard Error** | 0.735980072 | 0.801040989 | 0.683130051 | 0.584522597 | 0.376386326 | 0.801040989 |
| **Time Point** | **0 Hour** | | **3 Hour** | | **6 Hour** | |
|  | **Control** | **Lemon Eucalyptus** | **Control** | **Lemon Eucalyptus** | **Control** | **Lemon Eucalyptus** |
|  | 6 | 0 | 5 | 1 | 5 | 1 |
|  | 9 | 2 | 5 | 1 | 6 | 2 |
|  | 5 | 3 | 6 | 2 | 7 | 0 |
|  | 10 | 3 | 6 | 0 | 4 | 0 |
|  | 7 | 2 | 6 | 3 | 7 | 1 |
|  | 5 | 2 | 7 | 0 | 6 | 1 |
| Average | 7 | 2 | 5.833333333 | 1.166666667 | 5.833333333 | 0.833333333 |
| Standard Deviation | 2.097617696 | 1.095445115 | 0.752772653 | 1.169045194 | 1.169045194 | 0.752772653 |
| Standard Error | 1.048808848 | 0.547722558 | 0.376386326 | 0.584522597 | 0.584522597 | 0.376386326 |
| **Time Point** | **0 Hour** | | **3 Hour** | | **6 Hour** | |
|  | **Control** | **Picaridin** | **Control** | **Picaridin** | **Control** | **Picaridin** |
|  | 8 | 4 | 7 | 3 | 5 | 6 |
|  | 6 | 0 | 7 | 3 | 7 | 1 |
|  | 4 | 3 | 4 | 0 | 7 | 5 |
|  | 7 | 0 | 7 | 0 | 6 | 3 |
|  | 5 | 2 | 6 | 1 | 9 | 2 |
|  | 7 | 4 | 7 | 0 | 7 | 3 |
| **Average** | 6.166666667 | 2.166666667 | 6.333333333 | 1.166666667 | 6.833333333 | 3.333333333 |
| **Standard Deviation** | 1.471960144 | 1.834847859 | 1.211060142 | 1.471960144 | 1.329160136 | 1.861898673 |
| **Standard Error** | 0.735980072 | 0.91742393 | 0.605530071 | 0.735980072 | 0.664580068 | 0.930949336 |
| **Time Point** | **0 Hour** | | **3 Hour** | | **6 Hour** | |
|  | **Control** | **IR3535** | **Control** | **IR3535** | **Control** | **IR3535** |
|  | 5 | 4 | 9 | 3 | 5 | 4 |
|  | 5 | 4 | 5 | 1 | 5 | 4 |
|  | 10 | 4 | 8 | 4 | 8 | 2 |
|  | 6 | 1 | 5 | 3 | 7 | 3 |
|  | 6 | 2 | 7 | 2 | 5 | 3 |
|  | 7 | 3 | 6 | 4 | 6 | 3 |
| **Average** | 6.5 | 3 | 6.666666667 | 2.833333333 | 6 | 3.166666667 |
| **Standard Deviation** | 1.870828693 | 1.264911064 | 1.632993162 | 1.169045194 | 1.264911064 | 0.752772653 |
| **Standard Error** | 0.763762616 | 0.516397779 | 0.666666667 | 0.477260702 | 0.516397779 | 0.307318149 |

**Supplemental Table 1 – Raw data**

|  | **Number of Engagements** | | | **Standard Error** | | |
| --- | --- | --- | --- | --- | --- | --- |
| **Test** | **Initial** | **3 hr** | **6 hr** | **Initial** | **3 hr** | **6 hr** |
| CONTROL | 6.63 | 6.38 | 6.63 | 0.89 | 0.53 | 0.52 |
| DEET | 1.83 | 1.83 | 2.17 | 0.8 | 0.58 | 0.8 |
| OLE | 2 | 1.17 | 0.83 | 0.55 | 0.58 | 0.38 |
| Picaridin | 2.17 | 1.17 | 3.33 | 0.92 | 0.74 | 0.93 |
| IR3535 | 3 | 2.83 | 3.17 | 0.52 | 0.48 | 0.31 |

**Supplemental Table 2 – Average engagement numbers and standard errors.**

**Supplemental Figure 1- Data normality analysis.**

| **Tukey's multiple comparisons test** | **Mean Diff.** | **95.00% CI of diff.** | **Significant?** | **Summary** | **Adjusted P Value** |
| --- | --- | --- | --- | --- | --- |
|  |  |  |  |  |  |
| **Initial:Control vs. Initial:Deet** | **4.874** | **3.062 to 6.686** | **Yes** | ******** | **<0.0001** |
| **Initial:Control vs. Initial:OLE** | **4.852** | **3.040 to 6.664** | **Yes** | ******** | **<0.0001** |
| **Initial:Control vs. Initial:Picaridin** | **4.519** | **2.706 to 6.331** | **Yes** | ******** | **<0.0001** |
| **Initial:Control vs. Initial:IRH3535** | **3.407** | **1.595 to 5.219** | **Yes** | ******** | **<0.0001** |
| **Initial:Control vs. 3 hour:Control** | **0.2963** | **-0.9850 to 1.578** | **No** | **ns** | **>0.9999** |
| **Initial:Control vs. 3 hour:Deet** | **5.341** | **3.529 to 7.153** | **Yes** | ******** | **<0.0001** |
| **Initial:Control vs. 3 hour:OLE** | **5.519** | **3.706 to 7.331** | **Yes** | ******** | **<0.0001** |
| **Initial:Control vs. 3 hour:Picaridin** | **5.296** | **3.484 to 7.108** | **Yes** | ******** | **<0.0001** |
| **Initial:Control vs. 3 hour:IRH3535** | **3.963** | **2.151 to 5.775** | **Yes** | ******** | **<0.0001** |
| **Initial:Control vs. 6 hour:Control** | **0.08889** | **-1.192 to 1.370** | **No** | **ns** | **>0.9999** |
| **Initial:Control vs. 6 hour:Deet** | **4.519** | **2.706 to 6.331** | **Yes** | ******** | **<0.0001** |
| **Initial:Control vs. 6 hour:OLE** | **5.852** | **4.040 to 7.664** | **Yes** | ******** | **<0.0001** |
| **Initial:Control vs. 6 hour:Picaridin** | **3.185** | **1.373 to 4.997** | **Yes** | ******** | **<0.0001** |
| **Initial:Control vs. 6 hour:IRH3535** | **3.519** | **1.706 to 5.331** | **Yes** | ******** | **<0.0001** |
| **Initial:Deet vs. Initial:OLE** | **-0.02222** | **-2.242 to 2.197** | **No** | **ns** | **>0.9999** |
| **Initial:Deet vs. Initial:Picaridin** | **-0.3556** | **-2.575 to 1.864** | **No** | **ns** | **>0.9999** |
| **Initial:Deet vs. Initial:IRH3535** | **-1.467** | **-3.686 to 0.7527** | **No** | **ns** | **0.6111** |
| **Initial:Deet vs. 3 hour:Control** | **-4.578** | **-6.390 to -2.766** | **Yes** | ******** | **<0.0001** |
| **Initial:Deet vs. 3 hour:Deet** | **0.4667** | **-1.753 to 2.686** | **No** | **ns** | **>0.9999** |
| **Initial:Deet vs. 3 hour:OLE** | **0.6444** | **-1.575 to 2.864** | **No** | **ns** | **0.9996** |
| **Initial:Deet vs. 3 hour:Picaridin** | **0.4222** | **-1.797 to 2.642** | **No** | **ns** | **>0.9999** |
| **Initial:Deet vs. 3 hour:IRH3535** | **-0.9111** | **-3.130 to 1.308** | **No** | **ns** | **0.9852** |
| **Initial:Deet vs. 6 hour:Control** | **-4.785** | **-6.597 to -2.973** | **Yes** | ******** | **<0.0001** |
| **Initial:Deet vs. 6 hour:Deet** | **-0.3556** | **-2.575 to 1.864** | **No** | **ns** | **>0.9999** |
| **Initial:Deet vs. 6 hour:OLE** | **0.9778** | **-1.242 to 3.197** | **No** | **ns** | **0.9723** |
| **Initial:Deet vs. 6 hour:Picaridin** | **-1.689** | **-3.908 to 0.5304** | **No** | **ns** | **0.3652** |
| **Initial:Deet vs. 6 hour:IRH3535** | **-1.356** | **-3.575 to 0.8638** | **No** | **ns** | **0.732** |
| **Initial:OLE vs. Initial:Picaridin** | **-0.3333** | **-2.553 to 1.886** | **No** | **ns** | **>0.9999** |
| **Initial:OLE vs. Initial:IRH3535** | **-1.444** | **-3.664 to 0.7749** | **No** | **ns** | **0.6361** |
| **Initial:OLE vs. 3 hour:Control** | **-4.556** | **-6.368 to -2.743** | **Yes** | ******** | **<0.0001** |
| **Initial:OLE vs. 3 hour:Deet** | **0.4889** | **-1.730 to 2.708** | **No** | **ns** | **>0.9999** |
| **Initial:OLE vs. 3 hour:OLE** | **0.6667** | **-1.553 to 2.886** | **No** | **ns** | **0.9994** |
| **Initial:OLE vs. 3 hour:Picaridin** | **0.4444** | **-1.775 to 2.664** | **No** | **ns** | **>0.9999** |
| **Initial:OLE vs. 3 hour:IRH3535** | **-0.8889** | **-3.108 to 1.330** | **No** | **ns** | **0.9882** |
| **Initial:OLE vs. 6 hour:Control** | **-4.763** | **-6.575 to -2.951** | **Yes** | ******** | **<0.0001** |
| **Initial:OLE vs. 6 hour:Deet** | **-0.3333** | **-2.553 to 1.886** | **No** | **ns** | **>0.9999** |
| **Initial:OLE vs. 6 hour:OLE** | **1** | **-1.219 to 3.219** | **No** | **ns** | **0.9665** |
| **Initial:OLE vs. 6 hour:Picaridin** | **-1.667** | **-3.886 to 0.5527** | **No** | **ns** | **0.388** |
| **Initial:OLE vs. 6 hour:IRH3535** | **-1.333** | **-3.553 to 0.8860** | **No** | **ns** | **0.7544** |
| **Initial:Picaridin vs. Initial:IRH3535** | **-1.111** | **-3.330 to 1.108** | **No** | **ns** | **0.9233** |
| **Initial:Picaridin vs. 3 hour:Control** | **-4.222** | **-6.034 to -2.410** | **Yes** | ******** | **<0.0001** |
| **Initial:Picaridin vs. 3 hour:Deet** | **0.8222** | **-1.397 to 3.042** | **No** | **ns** | **0.9945** |
| **Initial:Picaridin vs. 3 hour:OLE** | **1** | **-1.219 to 3.219** | **No** | **ns** | **0.9665** |
| **Initial:Picaridin vs. 3 hour:Picaridin** | **0.7778** | **-1.442 to 2.997** | **No** | **ns** | **0.9968** |
| **Initial:Picaridin vs. 3 hour:IRH3535** | **-0.5556** | **-2.775 to 1.664** | **No** | **ns** | **>0.9999** |
| **Initial:Picaridin vs. 6 hour:Control** | **-4.43** | **-6.242 to -2.618** | **Yes** | ******** | **<0.0001** |
| **Initial:Picaridin vs. 6 hour:Deet** | **0** | **-2.219 to 2.219** | **No** | **ns** | **>0.9999** |
| **Initial:Picaridin vs. 6 hour:OLE** | **1.333** | **-0.8860 to 3.553** | **No** | **ns** | **0.7544** |
| **Initial:Picaridin vs. 6 hour:Picaridin** | **-1.333** | **-3.553 to 0.8860** | **No** | **ns** | **0.7544** |
| **Initial:Picaridin vs. 6 hour:IRH3535** | **-1** | **-3.219 to 1.219** | **No** | **ns** | **0.9665** |
| **Initial:IRH3535 vs. 3 hour:Control** | **-3.111** | **-4.923 to -1.299** | **Yes** | ******** | **<0.0001** |
| **Initial:IRH3535 vs. 3 hour:Deet** | **1.933** | **-0.2860 to 4.153** | **No** | **ns** | **0.1652** |
| **Initial:IRH3535 vs. 3 hour:OLE** | **2.111** | **-0.1082 to 4.330** | **No** | **ns** | **0.0811** |
| **Initial:IRH3535 vs. 3 hour:Picaridin** | **1.889** | **-0.3304 to 4.108** | **No** | **ns** | **0.194** |
| **Initial:IRH3535 vs. 3 hour:IRH3535** | **0.5556** | **-1.664 to 2.775** | **No** | **ns** | **>0.9999** |
| **Initial:IRH3535 vs. 6 hour:Control** | **-3.319** | **-5.131 to -1.506** | **Yes** | ******** | **<0.0001** |
| **Initial:IRH3535 vs. 6 hour:Deet** | **1.111** | **-1.108 to 3.330** | **No** | **ns** | **0.9233** |
| **Initial:IRH3535 vs. 6 hour:OLE** | **2.444** | **0.2251 to 4.664** | **Yes** | ***** | **0.0164** |
| **Initial:IRH3535 vs. 6 hour:Picaridin** | **-0.2222** | **-2.442 to 1.997** | **No** | **ns** | **>0.9999** |
| **Initial:IRH3535 vs. 6 hour:IRH3535** | **0.1111** | **-2.108 to 2.330** | **No** | **ns** | **>0.9999** |
| **3 hour:Control vs. 3 hour:Deet** | **5.044** | **3.232 to 6.857** | **Yes** | ******** | **<0.0001** |
| **3 hour:Control vs. 3 hour:OLE** | **5.222** | **3.410 to 7.034** | **Yes** | ******** | **<0.0001** |
| **3 hour:Control vs. 3 hour:Picaridin** | **5** | **3.188 to 6.812** | **Yes** | ******** | **<0.0001** |
| **3 hour:Control vs. 3 hour:IRH3535** | **3.667** | **1.855 to 5.479** | **Yes** | ******** | **<0.0001** |
| **3 hour:Control vs. 6 hour:Control** | **-0.2074** | **-1.489 to 1.074** | **No** | **ns** | **>0.9999** |
| **3 hour:Control vs. 6 hour:Deet** | **4.222** | **2.410 to 6.034** | **Yes** | ******** | **<0.0001** |
| **3 hour:Control vs. 6 hour:OLE** | **5.556** | **3.743 to 7.368** | **Yes** | ******** | **<0.0001** |
| **3 hour:Control vs. 6 hour:Picaridin** | **2.889** | **1.077 to 4.701** | **Yes** | ******** | **<0.0001** |
| **3 hour:Control vs. 6 hour:IRH3535** | **3.222** | **1.410 to 5.034** | **Yes** | ******** | **<0.0001** |
| **3 hour:Deet vs. 3 hour:OLE** | **0.1778** | **-2.042 to 2.397** | **No** | **ns** | **>0.9999** |
| **3 hour:Deet vs. 3 hour:Picaridin** | **-0.04444** | **-2.264 to 2.175** | **No** | **ns** | **>0.9999** |
| **3 hour:Deet vs. 3 hour:IRH3535** | **-1.378** | **-3.597 to 0.8415** | **No** | **ns** | **0.7089** |
| **3 hour:Deet vs. 6 hour:Control** | **-5.252** | **-7.064 to -3.440** | **Yes** | ******** | **<0.0001** |
| **3 hour:Deet vs. 6 hour:Deet** | **-0.8222** | **-3.042 to 1.397** | **No** | **ns** | **0.9945** |
| **3 hour:Deet vs. 6 hour:OLE** | **0.5111** | **-1.708 to 2.730** | **No** | **ns** | **>0.9999** |
| **3 hour:Deet vs. 6 hour:Picaridin** | **-2.156** | **-4.375 to 0.06377** | **No** | **ns** | **0.0668** |
| **3 hour:Deet vs. 6 hour:IRH3535** | **-1.822** | **-4.042 to 0.3971** | **No** | **ns** | **0.2437** |
| **3 hour:OLE vs. 3 hour:Picaridin** | **-0.2222** | **-2.442 to 1.997** | **No** | **ns** | **>0.9999** |
| **3 hour:OLE vs. 3 hour:IRH3535** | **-1.556** | **-3.775 to 0.6638** | **No** | **ns** | **0.5097** |
| **3 hour:OLE vs. 6 hour:Control** | **-5.43** | **-7.242 to -3.618** | **Yes** | ******** | **<0.0001** |
| **3 hour:OLE vs. 6 hour:Deet** | **-1** | **-3.219 to 1.219** | **No** | **ns** | **0.9665** |
| **3 hour:OLE vs. 6 hour:OLE** | **0.3333** | **-1.886 to 2.553** | **No** | **ns** | **>0.9999** |
| **3 hour:OLE vs. 6 hour:Picaridin** | **-2.333** | **-4.553 to -0.1140** | **Yes** | ***** | **0.0289** |
| **3 hour:OLE vs. 6 hour:IRH3535** | **-2** | **-4.219 to 0.2193** | **No** | **ns** | **0.1281** |
| **3 hour:Picaridin vs. 3 hour:IRH3535** | **-1.333** | **-3.553 to 0.8860** | **No** | **ns** | **0.7544** |
| **3 hour:Picaridin vs. 6 hour:Control** | **-5.207** | **-7.019 to -3.395** | **Yes** | ******** | **<0.0001** |
| **3 hour:Picaridin vs. 6 hour:Deet** | **-0.7778** | **-2.997 to 1.442** | **No** | **ns** | **0.9968** |
| **3 hour:Picaridin vs. 6 hour:OLE** | **0.5556** | **-1.664 to 2.775** | **No** | **ns** | **>0.9999** |
| **3 hour:Picaridin vs. 6 hour:Picaridin** | **-2.111** | **-4.330 to 0.1082** | **No** | **ns** | **0.0811** |
| **3 hour:Picaridin vs. 6 hour:IRH3535** | **-1.778** | **-3.997 to 0.4415** | **No** | **ns** | **0.281** |
| **3 hour:IRH3535 vs. 6 hour:Control** | **-3.874** | **-5.686 to -2.062** | **Yes** | ******** | **<0.0001** |
| **3 hour:IRH3535 vs. 6 hour:Deet** | **0.5556** | **-1.664 to 2.775** | **No** | **ns** | **>0.9999** |
| **3 hour:IRH3535 vs. 6 hour:OLE** | **1.889** | **-0.3304 to 4.108** | **No** | **ns** | **0.194** |
| **3 hour:IRH3535 vs. 6 hour:Picaridin** | **-0.7778** | **-2.997 to 1.442** | **No** | **ns** | **0.9968** |
| **3 hour:IRH3535 vs. 6 hour:IRH3535** | **-0.4444** | **-2.664 to 1.775** | **No** | **ns** | **>0.9999** |
| **6 hour:Control vs. 6 hour:Deet** | **4.43** | **2.618 to 6.242** | **Yes** | ******** | **<0.0001** |
| **6 hour:Control vs. 6 hour:OLE** | **5.763** | **3.951 to 7.575** | **Yes** | ******** | **<0.0001** |
| **6 hour:Control vs. 6 hour:Picaridin** | **3.096** | **1.284 to 4.908** | **Yes** | ******** | **<0.0001** |
| **6 hour:Control vs. 6 hour:IRH3535** | **3.43** | **1.618 to 5.242** | **Yes** | ******** | **<0.0001** |
| **6 hour:Deet vs. 6 hour:OLE** | **1.333** | **-0.8860 to 3.553** | **No** | **ns** | **0.7544** |
| **6 hour:Deet vs. 6 hour:Picaridin** | **-1.333** | **-3.553 to 0.8860** | **No** | **ns** | **0.7544** |
| **6 hour:Deet vs. 6 hour:IRH3535** | **-1** | **-3.219 to 1.219** | **No** | **ns** | **0.9665** |
| **6 hour:OLE vs. 6 hour:Picaridin** | **-2.667** | **-4.886 to -0.4473** | **Yes** | ****** | **0.0048** |
| **6 hour:OLE vs. 6 hour:IRH3535** | **-2.333** | **-4.553 to -0.1140** | **Yes** | ***** | **0.0289** |
| **6 hour:Picaridin vs. 6 hour:IRH3535** | **0.3333** | **-1.886 to 2.553** | **No** | **ns** | **>0.9999** |

**Supplemental Table 3 - Tukey's multiple comparisons test**


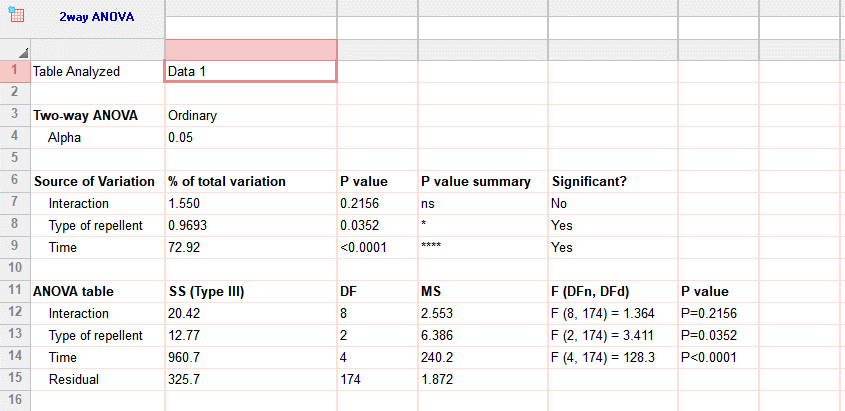


**Supplemental Table 4 – 2way ANOVA test**
